# Comment on the Effect of Endocannabinoid System on Rat Behavior

**DOI:** 10.15412/J.BCN.03080111

**Published:** 2017-01

**Authors:** Ali Roohbakhsh

I read the recently published article in Vol 6 (3) of Basic and Clinical Neuroscience entitled “Study the Effect of Endocannabinoid System on Rat Behavior in Elevated Plus-Maze” by Komaki et al. (2015). In this valuable article, the authors uncovered the effects of AM251 as a CB_1_ receptor antagonist on anxiety-like behaviors of rats using elevated plus-maze. However, I have a few comments on this article:

In the discussion section, the anxiogenic-like effect of AM251 in rats was completely attributed to blockade of CB_1_ receptors. However, based on the following argument, I believe the authors have ignored the role of GPR55 receptors in this process.

GPR55 receptors are a class of G protein-coupled receptors suggested as the third member of cannabinoid receptors family (Ryberg et al., 2007; Lauckner et al., 2008). Previous studies show that both 2-arachidonoylglycerol and anandamide (enhanced by URB597) are endogenous ligands for GPR55 receptors (Ryberg et al., 2007;Lauckner et al., 2008). Furthermore, AM251 is a potent GPR55 receptor agonist (Ryberg et al., 2007; Henstridge et al., 2010). On the other hand, CB_1_ and GPR55 receptors influence each other’s signaling pathways (Sharir et al., 2012; Kargl et al., 2012). The potential role of GPR55 receptors in anxiety has been evaluated recently (Rahimi, Hajizadeh Moghaddam, & Roohbakhsh, 2015). Accordingly, the intracerebroventricular administration of O-1602 and ML193 as GPR55 receptor agonist and antagonist produce anxiolytic- and anxiogenic-like effects in rats, respectively. Taken together, considering GPR55 receptors in the reported effects of AM251 and URB597 in the discussion, will improve our knowledge about the effects of these drugs on anxiety. One more comment, the researchers should be cautious in employing such drugs in future studies.

## References

[B1] Henstridge C. M. Balenga N. A. Schröder R. Kargl J. K. Platzer W. Martini L. (2010). GPR55 ligands promote receptor coupling to multiple signaling pathways. British Journal of Pharmacology, 160(3), 604–14. doi: 10.1111/j.1476-5381.2009.00625.x 20136841 PMC2931561

[B2] Kargl J. Balenga N. Parzmair G. P. Brown A. J. Heinemann A. Waldhoer M. (2012). The cannabinoid receptor CB1 modulates the signaling properties of the lysophosphatidylinositol receptor GPR55. Journal of Biological Chemistry, 287(53), 44234–248. doi: 10.1074/jbc.m112.364109 23161546 PMC3531739

[B3] Komaki A. Hashemi-Firouzi N. Shojaei Sh. Souri Z. Heidari S. Shahidi S. (2015). Study the effect of endocannabinoid system on rat behavior in elevated plus-maze. Basic and Clinical Neuroscience, 6(3), 147–154. 26904171 PMC4656987

[B4] Lauckner J. E. Jensen J. B. Chen H. Y. Lu H. C. Hille B. Mackie K. (2008). GPR55 is a cannabinoid receptor that increases intracellular calcium and inhibits M current. Proceedings of National Academy of Science USA, 105(7), 2699–704. doi: 10.1073/pnas.0711278105 PMC2268199 18263732

[B5] Rahimi A. Hajizadeh Moghaddam A. Roohbakhsh A. (2015). Central administration of GPR55 receptor agonist and antagonist modulates anxiety-related behaviors in rats. Fundamental and Clinical Pharmacology, 29(2), 185–90. doi: 10.1111/fcp.12099 25620584

[B6] Ryberg E. Larsson N. Sjögren S. Hjorth S. Hermansson N. O. Leonova J. (2007). The orphan receptor GPR55 is a novel cannabinoid receptor. British Journal of Pharmacology, 152(7), 1092–101. doi: 10.1038/sj.bjp.0707460 17876302 PMC2095107

[B7] Sharir H. Console-Bram L. Mundy C. Popoff S. N. Kapur A. Abood M. E. (2012). The endocannabinoids anandamide and virodhamine modulate the activity of the candidate cannabinoid receptor GPR55. Journal of Neuroimmunology and Pharmacology, 7(4), 856–865. doi: 10.1007/s11481-012-9351-6 PMC3669693 22454039

